# The Viewing Reaction Time as a Diagnostic Tool of Pedohebephilia in the Dunkelfeld

**DOI:** 10.1007/s10508-023-02662-y

**Published:** 2023-08-18

**Authors:** Till Amelung, Anna Konrad, Klaus M. Beier, Robert J. B. Lehmann

**Affiliations:** 1 Institut für Sexualwissenschaft und Sexualmedizin, Charité—Universitätsmedizin Berlin, Corporate Member of Freie Universität Berlin and Humboldt Universität zu Berlin, Luisenstraße 57, 10117 Berlin, Germany; 2 Klinik für Psychiatrie und Psychotherapie, CCM, Charité—Universitätsmedizin Berlin, Corporate member of Freie Universität Berlin and HumboldtUniversität zu Berlin, Klinik für Psychiatrie und Psychotherapie, CCM, Berlin, Germany; 3 Department of Psychology, Medical School Berlin, Berlin, Germany

**Keywords:** Pedophilia, Hebephilia, Viewing reaction time, Sexual preference, Sexual behavior, Dunkelfeld

## Abstract

Diagnosing pedohebephilia is fraught with obstacles given the tabooed nature of this sexual preference. The viewing reaction time effect (VRT) provides a non-intrusive indirect measure of sexual interest in minors. In forensic populations, the ability of the difference between the latencies while viewing child and adult sexual stimuli (VRT index) to discern child sexual offenders from a range of control groups has been ascertained meta-analytically. Given that the effect has been studied almost exclusively in forensic samples, its dependence or independence on prior overt (deviant) sexual behavior remains unclear. The present study sought to examine the relationship of prior sexual and non-sexual behaviors with the VRT in a sample of 282 self-referring, help-seeking men with and without pedohebephilia with and without a history of prior child sexual offenses (CSO) or a use of child sexual abuse materials (CSAM) recruited outside a forensic context. We found that (1) the clinical diagnosis of pedohebephilia but not prior CSO or CSAM showed a significant association with the VRT index; (2) the discriminatory ability of the VRT index did not differ significantly between samples with and without a history of prior overt sexual behavior with children; (3) the VRT index correlated positively with a behavioral marker of pedohebephilia in a subsample of individuals with prior judicially detected or undetected overt sexual behavior with children; and (4) in the same subsample, the VRT index correlated positively with markers of sexual interests in minors or hypersexuality but not of antisociality. Equivalence testing failed to refute a potential effect of prior sexual behavior on the VRT index. Our study showed that the VRT may provide an unintrusive diagnostic tool for pedohebephilia. The effect of prior overt sexual behavior with children needs further examination.

## Introduction

A persistent sexual interest in children manifesting through sexually arousing fantasies, sexual urges, or behaviors is the core feature of the clinical diagnosis of pedophilia (American Psychiatric Association, 2022; World Health Organization, 2020). Two representative studies in human males yielded estimates of 2.3–5.0% for any sexual interest and less than 0.1 to 0.6% for a genuine sexual preference for prepubertal children (Bártová et al., 2021; Dombert et al., 2016). In females, the only representative study of Bártová et al. (2021) found 0.4% of 5,021 women reporting any sexual interest in prepubescent children and 0% a genuine sexual preference. Seto (2017) argued that the sexual interest in prepubescent children can be considered a sexual orientation for age and proposes other chronophilias, such as hebephilia (i.e., sexual interest in early pubescent children, typically at ages 10 to 15 (Eckert-Lind et al., 2020; Kahl et al., 2007)). Stephens et al. (2017), however, found substantial overlap between indicators of pedophilia and hebephilia, including self-report, sexual behavior, and sexual arousal supporting the idea of pedohebephilia. In the DSM-5 and the ICD-11, only interests in children before puberty are subsumed under pedophilia or pedophilic disorder. However, given that hebephilic sexual interests have both physiological and behavioral correlates, and overlapping interests are common (Beier et al., 2013; Blanchard et al., 2008; McPhail et al., 2017), we will address sexual interest in minors as pedohebephilia.

Pedohebephilia is of specific public health concern not only given the potential consequences of its behavioral manifestations (e.g., child sexual offending; CSO) but also due to the stigmatization associated with this specific sexual interest. Not only does the social stigmatization of sexual interests in children not differentiate the interest and the actual sexual behavior (Jahnke et al., 2015). The associated stigma is even higher than for other paraphilias and psychological conditions linked to sexual offending (Lehmann et al., 2021). Non-offending individuals with pedohebephilia (minor attracted people; MAPs) may suffer from impairments in general psychological and social functioning due to their condition in addition to the perceived or imposed risk to children and thus be facing specific problems in living with this interest in terms of risk management and social functioning (Dymond & Duff, 2020; Walker, 2017). Accordingly, practitioners should understand that pedophilia (psychiatric disorder) and sexual offending against children (criminal behavior) are not equivalent (Seto, 2009, 2018). In the general population, research on the concordance between pedophilic fantasies and sexual victimization of children shows moderate effects (*r* = 0.48; Dombert et al., 2016) with the majority of men who indicated pedophilic fantasies reported no adult–child sexual behavior (56%). Jahnke (2018) pointed out that practitioners should learn to address issues associated with stigmatized sexual identities (e.g., deciding whether or not to reveal one’s sexual identity to others) as well as to be aware that stigma-related stress may exacerbate mental health problems and increase offending risk among people with pedophilia. Research in MAPs is scarce, given that this population has only recently begun to gain considerable attention. The “Dunkelfeld” projects in Germany offer treatment for self-referring individuals with self-identified sexual interests toward children outside a forensic setting (Beier et al., 2015, 2016). While in their origins the primary focus laid on the prevention of child sexual offending, more recent developments have included additional efforts to manage stigma-related and other distress (Konrad, 2021)**.**

In samples of convicted CSO, roughly half do not show clinical signs of pedohebephilia (Seto, 2018). In offenders convicted for using child sexual abuse material (CSAM; (Greijer & Doek, 2016)), a similar dissociation of the behavior and the sexual preference appears to hold true, too, though research points at a greater prevalence of pedohebephilic sexual interest in CSAM offenders and CSAM has been proposed to be a marker of pedophilic interests (Seto, 2018; Seto et al., 2006). Showing overt behavior in terms of child sexual offenses has been included in the core psychopathology of pedophilic disorders (American Psychiatric Association, 2000). For treatment and prognosis, however, it is important to consider that showing the overt behavior does not necessarily imply that this behavior was driven by a sexual interest in children (Seto, 2018).

In a clinical and forensic context, a reliable diagnosis of sexual interest in children is thus of paramount importance for planning and conducting both preventive therapy for child sexual offending and treatment for individuals concerned about their sexual preferences.

### Diagnosing Sexual Interest in Children

The most direct way to assess pedophilic interest in clinical assessment is via self-report or questionnaires. For diagnosing pedophilia according to the DSM-5, questions about sexual interest need to be accompanied by questions about persistence, recurrence, intensity, and duration (Seto, 2018). However, sexual interest in children in a clinical context may not be easily ascertained. The substantial stigmatization of this interest as well as potential judicial consequences of associated behaviors may preclude affected individuals to admit to any such fantasies or urges and hinder the diagnosis through direct exploration. This obstacle not only complicates clinical assessment potentially resulting in low inter-rater reliability for the clinical diagnosis of pedophilia (Mokros et al., 2018) but also impairs research. Given the problems with the reliability of pedophilia diagnosis, Marshall and Kingston (2018) argued for alternative diagnostic strategies.

Single, distinguishable characteristics of individual acts of adult/child sexual behavior have been found to be correlated with diagnosable sexual interest in children (Lehmann et al., 2018; Seto & Lalumière, 2001). Diagnostic rating scales such as the screening scale for pedophilic interest (SSPI and SSPI-2 in its second version) assess victim-related offense characteristics known to be associated with pedophilia such as one male victim, multiple victims, victims under the age of 12, and extrafamilial victims, and the use of CSAM (Seto et al., 2015). Higher scores on this scale have been found to be correlated with sexual re-offenses against children as well as with a psychophysiological marker of sexual interest in children (phallometry, see also below) (Helmus et al., 2015).

Besides self-report and diagnostic rating scales, psychophysiological measures can add relevant information to the assessment of sexual interest, which can be considered the most direct way to measure male sexual response in the laboratory. Phallometry describes the measuring of penile responses to sexually salient stimuli and is thought to be the gold standard for the psychophysiological diagnosis of pedohebephilia in men (McPhail et al., 2017). However, there are various problems that remain with this procedure regarding the standardized laboratory setups, stimulus sets, and interpretation of results among different laboratories. Furthermore, the assessment is time-consuming, expensive, and intrusive. Especially in a non-forensic setting where self-referring individuals open up about their tabooed sexual preferences sometimes for the first time in their lives, the procedure of strapping one’s penis to laboratory appliances has the potential of endangering the vulnerable relationship between client and therapist. In summary, “under adversarial conditions, not everyone will cooperate with the testing protocol or produce interpretable results” (Thornton et al., 2018). A recent international overview shows that phallometric assessment is rarely used outside of Canada and the USA (Bickle et al., 2021).

Indirect, latency-based measures are considered less uncomfortable. The best established and most studied approach is called the viewing reaction time (VRT). The approach dates back 81 years (Rosenzweig, 1942) when recorded response latencies while watching sexually salient visual stimuli were shown to discern individuals with schizophrenia with “high” and “low” frequency sexual behavior. In the context of adult child sexual interactions, stimuli of children have been confirmed meta-analytically to elicit longer response latencies than stimuli of adults in child sexual offenders compared with community controls or other offender groups (Schmidt et al., 2017). The same meta-analysis, however, found only small correlations with other physiological or behavioral markers of pedophilic sexual interests such as self-report (*r* = 0.38), the SSPI (*r* = 0.21), and phallometry (*r* = 0.25) with some of the aggregated studies showing zero or even negative correlations (Schmidt et al., 2017). In addition, research indicates VRT to be correlated with risk of recidivism as assessed by static, but not dynamic risk factors (Babchishin et al., 2013; Schmidt et al., 2017). That is, while the data suggest a correlation of the VRT with pedophilic sexual interest, its extent may be small. Given the forensic samples examined, factors associated with prior adult child sexual behavior might better explain the robust difference in response latencies. In fact, research on the VRT in teleiophilic individuals, i.e., persons exclusively attracted to mature body types, suggests that the process causing the VRT effect cannot be understood as a mere correlate of sexual arousal (Imhoff et al., 2012). Rather than through affective or attentional effects elicited by the visual stimuli, the VRT effect in teleiophilic populations appears to depend in large parts on the task and on voluntary evaluations of the stimuli (Imhoff et al., 2010, 2012; Pohl et al., 2016). Similar examinations in individuals with pedohebephilia are missing.

One possibility to examine the potential dependence of the effect on prior behavior lies in comparing non-offending and offending individuals with pedohebephilia. For the VRT, only one such examination has been conducted in two online samples of self-identifying individuals with pedohebephilia (Jahnke et al., 2022). This study found no differences in the VRT effect between individuals with pedohebephilia who self-reported prior convictions for sexual offenses including CSAM offenses and those who did not. Their study, however, did not control for undetected or unconvicted, i.e., Dunkelfeld sexual offenses and their criteria for pedophilia and hebephilia were based on self-report alone. Thus, a thorough examination of the potential influence of overt adult/child sexual behavior on the VRT effect in individuals with clinically ascertained pedohebephilia remains missing.

### Current Study

Given the problems with self-report data, phallometric assessment and sexual behavior (e.g., SSPI) being not applicable in samples of MAPs, the VRT provides an important diagnostic tool for the assessment of pedohebephilic sexual interest in a clinical, non-forensic therapeutic context. However, data assessing the applicability of this approach outside forensic populations is scarce. The present study thus sought to analyze the convergent and divergent validity of the VRT effect in a Dunkelfeld setting. We examined the dependency or independence of the VRT effect from prior overt sexual behavior by analyzing a sample of offending and non-offending individuals with and without clinically diagnosable pedohebephilia. In this regard, we were interested in whether the VRT is an indicator of sexual interest or sexual behavior.

In a sample of self-referring, help-seeking individuals in a non-forensic clinical setting, we expected convergence between indicators of sexual interest in children (clinical diagnosis, VRT) independent of sexual behavior with similar diagnostic accuracy in individuals with and without a history of child sexual offenses.

In individuals with prior sexual offenses, we investigated the convergent validity between VRT and sexual behavior using the SSPI-2 diagnostic rating scale. In addition, we were interested in the relationship between VRT and risk scales. Specifically, we expected a stronger relationship with factors indicative of deviant sexual interest (i.e., convergent validity) than indicators of (antisocial) behavior (i.e., divergent validity).

We deduced four hypotheses for the scope of the present study:

- Hypothesis 1: Clinically diagnosed pedohebephilia shows a significant, positive association with a VRT index regardless of self-reported prior CSO or CSAM.
- Hypothesis 2: The VRT index separates pedohebephilic and non-pedohebephilic individuals with similar accuracy in those with or without a prior history of sex offenses.
- Hypothesis 3: In self-referring individuals with self-reported prior CSO, the VRT index shows significant positive correlations with behavioral markers of deviant sexuality motivation (i.e., SSPI-2).
- Hypothesis 4: In self-referring individuals with self-reported prior CSO, the VRT index shows significant positive correlations with recidivism risk (i.e., an adapted version of the Static-99R, STABLE-2007).
- Hypothesis 4a: Specifically, we expect stronger correlations with items assessing deviant sexual interest than items assessing (antisocial) behavior.

## Method

### Sample

Subjects for the present study were recruited via a therapeutic program aiming at individuals with a sexual interest in children outside the judicial system. Between August 17, 2012 and March 29, 2018, *n* = 740 individuals applied for treatment within the program. Of these, *n* = 154 were ineligible for the present study due to non-male sex (*n* = 8), missing clinical data (*n* = 47), uncertainty of clinical diagnosis of sexual preference (*n* = 104), or uncertainty of clinical diagnosis of past child sexual offenses (*n* = 59), leaving *n* = 586 eligible for the analysis.

**Fig. 1:**
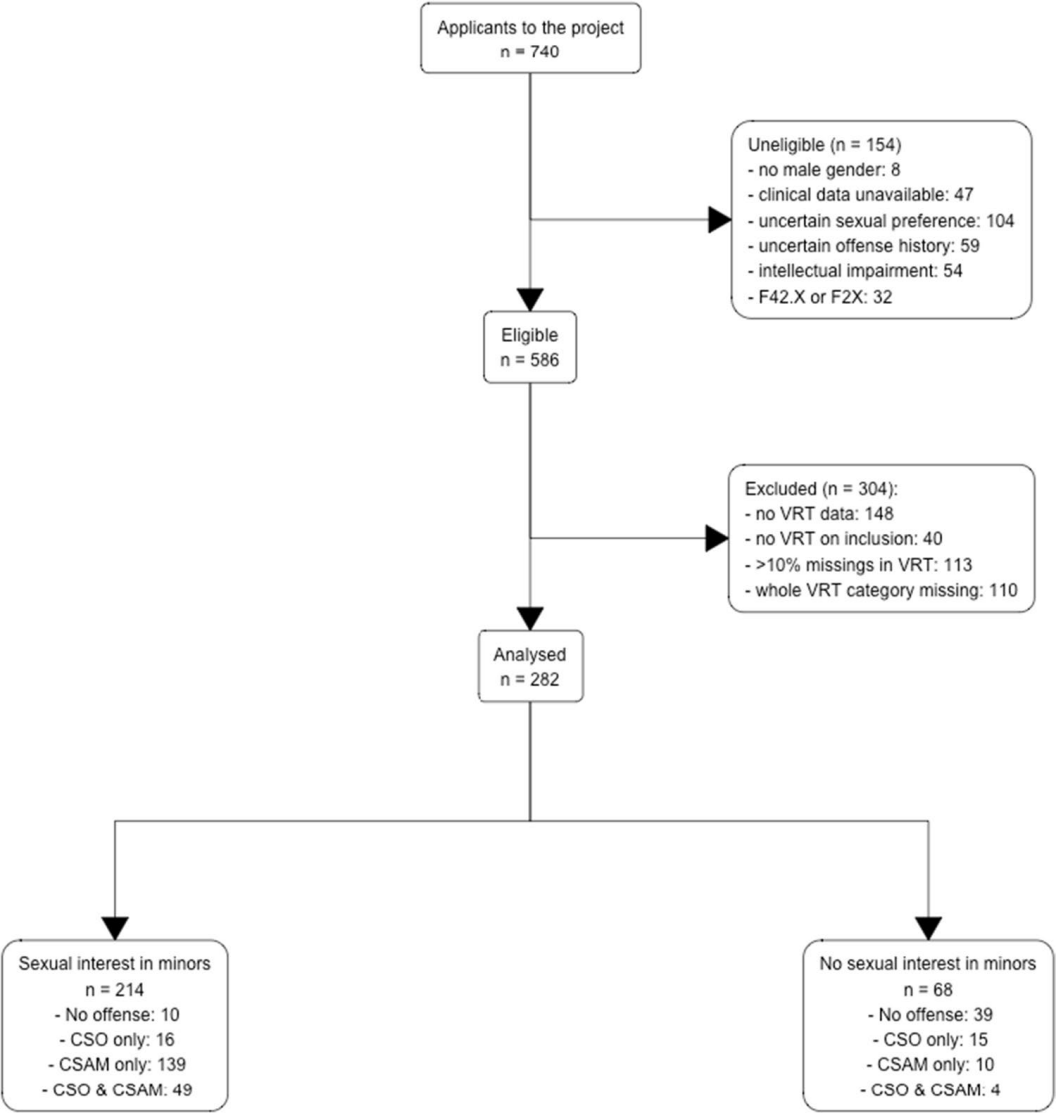
Flowchart illustrating the composition of the included sample. CSO: Child sexual offenses; CSAM: Child sexual abuse material offenses; VRT: Viewing reaction time; F42.X: ICD-10 Obsessive compulsive disorder; F2X: ICD-10 Psychotic disorder. Reasons for ineligibility and exclusion are not mutually exclusive

Of all eligible participants, *n* = 304 were excluded from the analyses if VRT data were absent (*n* = 148). Reasons for not performing the VRT task were not systematically assessed but could comprise refusal of completion by the client, neglect by the examiner, or technical problems. As our knowledge of the stability of the VRT effect over time in individuals with pedohebephilia is limited, individuals were also excluded if VRT testing had been performed more than four weeks apart from the clinical interview (*n* = 40). Our VRT paradigm allows for missing values. Missing values had thus to be imputed (see section Missing values imputation below). The risk of introducing systematic bias through imputation increases with the number of imputed values. There seem to be no empirically established thresholds balancing this risk of bias (Dong & Peng, 2013). We thus chose a threshold based on both pragmatic (retained sample size) and empirical (Bennett, 2001) considerations of 10% leading to the exclusion of 113. We further excluded individuals who showed completely missing values for at least one age and sex category (*n* = 110). Note that criteria for eligibility and inclusion were not mutually exclusive. The sample included for further analysis comprised 214 men with a sexual interest in minors and 68 men with no sexual interest in minors (see also Fig. 1). Individuals without a sexual interest in minors presented for a number of reasons including CSO and CSAM offenses motivated by other factors than a pedohebephilic sexual preference, or other uncertainties of their sexual preference (e.g., other paraphilias).

Excluded participants showed higher frequencies of prior conviction of CSO or CSAM and lower frequencies of sexual interest in early pubescent minors but were otherwise similar concerning sociodemographic and clinical characteristics (see Table 1). This difference reflects the program’s target population of individuals outside the judicial system. The focus of the program leads to declining further support within the program for individuals under judicial supervision but instead to defer these individuals to other specialized institutions. Individuals under judicial supervision thus often either refuse further examination or are offered to be spared the potentially distressing procedure given their unrelatedness to the clients’ looking for help. As our pre-analytic decisions may have had an influence on the study’s results, all planned analyses were rerun including individuals excluded for considerations of data quality. Also, analyses were rerun using raw VRT values. Detailed results can be accessed in Supplements one and two.

**Table 1:** Sociodemographic, psychiatric, and forensic characteristics of included and excluded individuals

	Excluded n = 304	Included n = 282	*t*	*p*
	M (SD)	M (SD)		
Age	38.39 (12.57)	39.17 (13.21)	− 0.7264	0.4679
STATIC-PPD (*n* = 65, range 0–8)	–	1.95 (1.87)		
STABLE-2007 (*n* = 49, range 2–20)	–	8.73 (4.22)		
ACUTE (*n* = 49, range 0–14)	–	4.33 (2.5)		
SSPI (*n* = 54, range 0–5)	–	3.02 (1.49)		
STATIC Paraphilia (*n* = 65, range 0–6)	–	1.75 (1.67)		
STATIC Youthful Stranger Aggression (*n* = 65, range 0–3)	–	0.71 (0.88)		
STATIC General Criminality (*n* = 65, range 0–4)	–	0.38 (0.78)		
STABLE Antisociality (*n* = 48, range 0–11)	–	4.02 (3.04)		
STABLE Sexual Deviance (*n* = 47, range 1–4)	–	2.23 (0.67)		
STABLE Hypersexuality (*n* = 48, range 0–4)	–	2.1 (1.13)		

	N (%)	N (%)	*χ*^*2*^	*p*
> 10 yrs education	153.0 (51%)	141 (50%)	0.01	0.9429
Custodian for kids	102 (34%)	107 (38%)	0.93	0.3352
Employed	218 (72%)	214 (77%)	1.06	0.2197
In a relationship	126 (41%)	127 (45%)	0.69	0.4061
Any known CSO	108 (36%)	84 (30%)	1.93	0.1643
Any known CSAM	190 (62%)	202 (72%)	5.10	0.0239
No known CSO or CSAM	71 (23%)	49 (17%)	2.86	0.0911
Any known CSAM convictions	66 (22%)	33 (12%)	9.74	0.0018
Any known CSO convictions	46 (15%)	24 (9%)	5.48	0.0192
Any other known delinquency	30 (10%)	31 (11%)	0.10	0.7566
Any additional psychiatric diagnosis	117 (48%)	79 (38%)	3.67	0.0554
Sexual interest in adults^✝^	250 (82%)	231 (82%)	0.00	1
Sexual interest in minors	205 (35%)	214 (37%)	4.72	0.0298
Sexual interest in early pubescent minors^✝✝^	166 (55%)	181 (64%)	5.17	0.0230
Sexual interest in prepubescent minors^✝✝^	114 (38%)	125 (44%)	2.55	0.1105

^✝^Including individuals with non-exclusive pedohebephilic preferences

^✝✝^including individuals with both pedophilic and hebephilic sexual interests

STATIC-PPD, STABLE-2007, and ACUTE were assessed only in included individuals with pedohebephilia who reported prior child sexual offenses. Differing missing values between sum scores and facettes were due to single missing items

### Procedure

Data were gathered as part of the intake procedure of the program. Applicants anonymously contacted the program and were invited to a clinical examination to ascertain sexual interests toward prepubescent and/or early pubescent body schemes, risk for offending, and treatment need. On the date of the examination, participants gave written informed consent into the assessment procedure and the use of their data for future publication anonymously. Following consent, applicants underwent a clinical interview to gather data on sexual urges, sexual fantasies capable of eliciting orgasm, and sexual behavior including pornography use and sexual offenses against minors and adults, legal sexual encounters, romantic and courtship behavior, prior convictions, and medical and mental health history. Interviews lasted between 90 and 180 min. Pauses were granted as needed. Following the interview, participants took the viewing reaction time test before additional questionnaire testing.

### Viewing Reaction Time Testing

The viewing reaction time test was presented on two different computers, a laptop and a tablet PC, running Windows XP and Neurobehavioral Systems’ Presentation® software that were administered by a trained research assistant. Before the actual testing, an instruction screen was presented in German indicating the response keys and the task. The task was described as to rate sexual attractiveness of the presented stimuli as quickly as possible on a 4-point Likert scale from “not at all sexually attractive” to “very sexually attractive.” Responses on both the laptop and the tablet PC were entered using a standard computer keyboard using the middle and the index fingers of both hands on the number keys “5” to “8.” Participants were free to read the instructions at their own pace and had the opportunity to clarify open questions with the research assistant, before they were left alone in the examination room to complete the task. The stimuli presented in the VRT consisted of nude images from the “Not Real People Set” (Pacific Assessment, Victoria, BC). The Not Real People Set consists of 80 images of bodies where faces and heads of other persons were mounted onto the image through computer software. The stimuli comprise males and females in developmental stages one through five after Tanner (1973), where Tanner stage 1 represents the prepubertal, Tanner stages 2 and 3 pubertal, Tanner stage 4 late pubertal/early postpubertal, and Tanner stage 5 the mature developmental age. Stimuli were presented in random order, preceded by a fixation cross in the middle of the computer screen of 2000 ms duration. The stimulus was removed from the screen either after participants had entered an attractiveness rating or after 5000 ms. The paradigm recorded both the attractiveness rating given and the time elapsed between first presentation of the stimulus and the response. In case the participant failed to provide an answer within 5000 ms, the trial was recorded as missing. Summary statistics of reaction times and attractiveness ratings are given in Table 2. To control for the group differences in general reaction time, raw viewing reaction time data were ipsatized per individual. The mean ipsatized reaction times per age and sex category were calculated. An index (VRT index) was computed as the difference between the maximum mean ipsatized reaction time toward any category of pre- and peripubertal stimuli (males or females in Tanner stages 1 through 3) minus the maximum mean ipsatized reaction time toward any category of postpubertal stimuli (males or females in Tanner stages 4 and 5). This approach was used to avoid null effects due to different target sex of the individuals’ sexual orientation as have been found before (Banse et al., 2010). For example, homosexual teleiophilic individuals were expected to produce the longest mean viewing time latencies in stimulus categories of adult males and the shortest in stimulus categories of adult females, whereas in heterosexual teleiophilic individuals this effect was expected to be reversed. Aggregating viewing time latencies of all adult stimuli combined were expected to then lead to values of roughly the average viewing time latency, attenuating potential differences. A similar argument can be construed for pedohebephilic individuals regarding response time latencies toward pre- and peripubertal stimulus categories of different sexes.

**Table 2:** Description of the viewing reaction time data in the final sample

	No sexual interest in minors (n = 68)	Any sexual interest in minors (n = 214)	*t*	df	*p*
Mean overall viewing reaction time	1333 (426)	1700 (518)	5.85	135	***
Mean reaction time MT5	1161 (572)	1191 (554)	1.96	109	
Mean reaction time MT4	1126 (523)	1390 (691)	− 1.20	128	
Mean reaction time MT3	1098 (490)	1460 (777)	− 6.85	123	***
Mean reaction time MT2	994 (456)	1433 (786)	− 9.16	146	***
Mean reaction time MT1	1005 (429)	1426 (741)	− 9.65	162	***
Mean reaction time FT1	1154 (516)	1932 (743)	− 5.81	198	***
Mean reaction time FT2	1217 (568)	2001 (742)	− 5.69	198	***
Mean reaction time FT3	1379 (690)	2053 (759)	− 4.55	181	***
Mean reaction time FT4	1999 (651)	2112 (749)	− 3.34	147	**
Mean reaction time FT5	2201 (757)	1996 (722)	− 0.39	110	
Raw reaction time index (max T1-3)—(max T4/5)	− 820 (659)	98 (558)	− 10.36	99	***
Mean attractiveness rating MT5	1.21 (0.6)	1.12 (0.46)	2.20	116	*
Mean attractiveness rating MT4	1.21 (0.57)	1.3 (0.58)	− 5.16	141	***
Mean attractiveness rating MT3	1.11 (0.43)	1.41 (0.71)	− 11.92	224	***
Mean attractiveness rating MT2	1.09 (0.39)	1.47 (0.76)	− 12.06	247	***
Mean attractiveness rating MT1	1.07 (0.38)	1.36 (0.64)	− 10.93	253	***
Mean attractiveness rating FT1	1.08 (0.38)	1.9 (0.87)	− 4.66	196	***
Mean attractiveness rating FT2	1.09 (0.4)	2.01 (0.87)	− 5.35	222	***
Mean attractiveness rating FT3	1.14 (0.44)	2.08 (0.85)	− 4.15	191	***
Mean attractiveness rating FT4	1.74 (0.64)	2.23 (0.8)	− 1.14	115	
Mean attractiveness rating FT5	2.26 (0.82)	2.01 (0.85)	1.21	93	

*M* male stimuli, *F* female stimuli, *T* developmental stage according to Tanner (1974)

Reaction times are given in milliseconds. Attractiveness ratings were given on a Likert scale from 1 “not at all sexually attractive” to 4 “very sexually attractive.” All comparisons computed in raw data without imputation. Different degrees of freedom reflect single missing values in any given category per individual

*p < .05; **p < .01; ***p < .001

### Clinical Diagnosis of Pedophilia

A clinical diagnosis of pedohebephilia was ascertained using a multi-step procedure. First, the clinician conducting the interview gave a first suspected diagnosis based on ICD-10 criteria for Pedophilia (i.e., including early pubertal stages). An independent rater then rated the information given in the notes taken by the clinician concerning the content of sexual fantasies and behaviors and gave an estimate of the probable sexual preference, i.e., pedohebephilia or teleiophilia, homo-, hetero-, or bisexual. In cases of uncertainties, missing information, or diverging clinical impression, interviewer and rater conferred to reach an agreement. If no agreement was possible, the case was marked as uncertain and further steps to ascertain the clinical diagnosis were initialized within the context of the treatment program (further examination, third-party anamnesis, or other). Such uncertain cases were removed from the present analysis. Note that the steps for ascertaining the clinical diagnosis described above were performed independently of the VRT data.

### Clinical Diagnosis of Child Sexual Offenses and Child Sexual Abuse Materials

Given that the program guaranteed anonymity to its participants, official police records were unavailable to confirm the self-reported prior offenses. Accordingly, a similar approach as described above was chosen to ascertain CSO and CSAM, i.e., clinical interview data were rated by two independent raters and re-evaluated in case of doubt. Child sexual offending was identified whenever an individual admitted to at least one sexual offense against children under the age of 14, the legal age of consent in Germany. Sexual offenses were defined as sexually touching or manipulating a child’s naked body, penetrating a child, making a child touch or manipulate the offender’s genitals or penetrate him, or sexually interacting with a child by showing pornography or using digital communication media. Offenses involving CSAM were ascertained given the individual reported the use of images or videos depicting children in prepubertal or pubertal developmental stages in above described sexual acts performed by or on them or in their presence, in a fully or partially undressed state in sexual poses or with a focus on the bare genitalia or buttocks. The classification relied on the scale developed within the COPINE-project and represented levels five to ten (Taylor et al., 2001).

### External Criteria for Pedophilia and Sexual Offense Risk

In individuals with pedohebephilia where prior CSO was established clinically, additional measures from the sex offender literature were applied to examine the external validity of the VRT in the Dunkelfeld.

*Screening Scale for Pedophilic Interest—2 (SSPI-2)* (Seto et al., 2015). The SSPI-2 is a five-item rating scale to assess the probability of pedophilic interests in convicted sexual offenders against children. The items are scored as yes/no decisions on the offense history and comprise any male victim, any victim under the age of 12, any extrafamilial victim, multiple victims, and any known child pornography offenses. The simple sum score of the scale was positively correlated with measures of penile response to visual sexual stimuli and re-offense risk (Seto et al., 2017a,b). The SSPI-2 has not been validated for the Dunkelfeld yet.

*STATIC-99-Dunkelfeld.* For the purpose of this study, the German version of the STATIC-99 (Eher et al., 2011) was adapted to be used in the Dunkelfeld. The STATIC-99 is an actuarial risk assessment tool developed in convicted sexual offenders against children (Harris et al., 2003). It assesses ten “static,” i.e., unchangeable life history variables known to influence re-offense risk, including (1) age at release of the offender, (2) ever lived with an intimate partner for more than two years, (3) any separate non-sexual violent offense at the same time of the index-offense, (4) prior non-sexual violent offenses, (5) number of prior sex offenses, (6) number of prior sentencing dates, and (7) any convictions for non-contact sex offenses, (8) any unrelated victims, (9) any stranger victims, and (10) any male victims. Research identified three factors underlying the overall measure: “Paraphilia,” represented through prior sex offenses, non-contact sexual conviction, male victim, two or more young victims, and unrelated victims; “Youthful stranger aggression,” represented by reversed age at release, never lived with an intimate partner for more than two years, index non-sexual violence and unrelated/stranger victims; and “General criminality,” represented by any prior involvement with the justice system, prior sentencing, prior non-sexual violence, prior supervision breaches and years free prior to index sex offense (Brouillette-Alarie et al., 2016). The STATIC-99 has thus far not been validated in the Dunkelfeld.

As many of the items rely on some “index-offense,” i.e., an offense leading to the assessment, the tool is not easily transferred to a situation where the examinees seek help self-motivatedly and not necessarily related to a recent sexual offense. We thus changed the coding of items to better suit the context of the assessment. The item assessing non-sexual violence involved in the index-offense was dropped completely. Age at assessment was used instead of age at release. The remaining eight items were rated independently of any “index-offense” by three independent raters using the clinical documentation. Complete scores for *n* = 64 individuals were gathered that way. Given our adaptation of the instrument for the Dunkelfeld, the factors “Youthful stranger aggression” and “General criminality” had to be altered slightly. Our factor “Youthful stranger aggression” was computed as the sum of age at assessment < 25, never lived with an intimate partner for more than two years, prior non-sexual violence, and unrelated/stranger victims. “General criminality” was represented by the number of prior convictions and prior non-sexual violence.

Items of the SSPI-2 and the STATIC-99-Dunkelfeld were rated from clinical files by three independent raters (TA, SE, and CJ). For twelve individuals, all items were coded by all three raters to compute intraclass correlation coefficients (ICC). In these, three items showed hardly any variance at all, yielding no interpretable ICC (prior conviction for non-sexual violence all zeros except for one rater rating 1, any stranger victims all zeros except for 1 case rated NA by two of three raters, prior non-contact sexual offenses all zeros except for two cases rated 1 by one rater each). The remaining ICCs ranged from 0.61 to 0.96 (median 0.88). Details are given in Table 3.

**Table 3:** Intraclass correlation coefficients for adapted STATIC-99-R and SSPI-2 items

		95% confidence interval	
	ICC	Lower bound	Upper bound
Prior convictions	0.61	0.35	0.82
Male victim	0.77	0.57	0.90
Prior convictions for non-sexual violence	n.a		
Relationship > 2 years	0.83	0.66	0.93
multiple victims	0.67	0.43	0.85
Victim known > 24 h	n.a		
Stranger victim	0.34	0.05	0.65
Victim < 12 years of age	0.89	0.78	0.96
Prior convictions for non-contact sexual offenses	n.a		

*ICC* Intraclass correlation coefficient calculated as the “classical,” one-way random effect model *ICC. n.a.* ICC not calculable due to zero variance

*STABLE-2007* (Hanson, 2007)*.* The STABLE-2007 is a scale to allow structured clinical assessment of dynamic risk factors for sexual offending in adult male sexual offenders. The STABLE-2007 is meant to be rated following an interview of the offender, in which the risk items have been explored by a trained, experienced rater. The 13 risk items include (1) significant social influences, (2) capacity for relationship stability, (3) emotional identification with children, (4) hostility toward women, (5) general social rejection/loneliness, (6) lack of concern for others, (7) impulsive acts, (8) poor cognitive problem solving, (9) negative emotionality and hostility, (10) sex drive/preoccupation, (11) sex as coping, (12) deviant sexual interest, and (13) cooperation with supervision. Research has identified three factors underlying the overall measure: “Antisociality,” represented by reversed capacity for relationship stability, hostility toward women, general social rejection/loneliness, lack of concern for others, impulsive acts, poor cognitive problem solving, and negative emotionality/hostility; “Sexual deviance,” represented by emotional identification with children and deviant sexual interests; and “Hypersexuality,” represented by sex drive/preoccupation and sex as coping (Etzler et al., 2020). The measure exists in a translated and validated German version (Etzler et al., 2020; Fernandez et al., 2012). Though not originally designed for the Dunkelfeld, the items can be readily transferred to a clinical situation outside a forensic setting. A formal validation of the STABLE-2007 in the Dunkelfeld, however, is missing.

*ACUTE-2007* (Hanson, 2007)*.* The ACUTE-2007 is an instrument designed to assess recent, risk-relevant behavior of sexual offenders in the community. Rating follows an interview by a trained, experienced clinician. The seven items rated comprise (1) access to a potential victim, (2) hostility, (3) sexual preoccupation, (4) rejection of supervision, (5) emotional collapse, (6) change in social support, and (7) substance abuse. The measure exists in a translated German form (Rettenberger & Matthes, 2008). The items of the ACUTE-2007 can readily be transferred into the Dunkelfeld setting. A formal validation in the Dunkelfeld is missing.

Descriptive data for all sum scores and facets of the external criteria are given in Table 1.

### Missing Values Imputation

With missing values allowed by the VRT paradigm, we planned a sensitivity analysis to compare the effects of possible imputation methods. As the main goal of our analysis was to provide an understanding of how applicable the VRT is in self-referring pedophebephilic individuals, we chose the simplest and thus most readily available method of missing values imputation as a reference point, i.e., replacing the missing value with the mean of the reaction times of the individual within the same age and sex category. Additional methods to impute missing values included predictive mean matching per age and sex category with and without individuals as separate classes and whole sample linear regression per age and sex category. All imputations were calculated using the “Multivariate Imputation by Chained Equations, mice” package, version 3.11.0 in R statistical software version 3.6.2 (https://www.R-project.org/). Individual mean and linear regression were performed as simple imputations, whereas probability mean matching was performed as multiple imputations yielding five imputed datasets each. For multiple imputations, results were combined according to Rubin’s rules (Barnard & Rubin, 1999; Rubin, 1987).

### Statistical Analysis

Hypothesis 1 was tested using linear regression of the VRT index on dummy coded variables for clinically diagnosed pedophilia, self-reported prior CSO, self-reported prior CSAM, and their two- and three-way interactions. Inspection of residuals was used to determine outliers and independence of residuals. Our Hypothesis 1 thereby needed to be viewed as a conjoint hypothesis stating both a significant positive association of the VRT index with clinically diagnosed pedohebephilia and negligible effects of prior overt sexual behavior with children on this association. The first statement can readily be tested following a classical null-hypothesis statistical testing (NHST) logic. For the examination of the second statement of “negligible effects of prior overt sexual behavior with children,” however, one must keep in mind that in the NHST logic, the rejection of the alternative hypothesis must not be considered as proof of the null hypothesis. Put otherwise, a statistically nonsignificant influence of prior overt sexual behavior with children on the association of the VRT index with clinically diagnosed pedohebephilia (i.e., statistically nonsignificant β-estimates of the interaction terms in our linear regression model) cannot be assumed to speak for the absence of such influence. In fact, in NHST the alpha constitutes a probability threshold below which we can only assume that the measured value is too unlikely to occur under the null hypothesis. If the test statistics result in a probability value greater than alpha, “the only formally correct conclusion is that the data are not surprising, assuming the null hypothesis is true” (Lakens, 2017).

The adequate statistical technique to determine whether an effect is too small to be considered relevant is to apply equivalence tests. For an equivalence test, a lower and an upper boundary, the smallest effect sizes of interest (SESOI), have to be determined that constitute the range of a negligible effect. Two composite null hypotheses can then be tested, i.e., that the measured effect is greater than the upper and smaller than the lower boundary. When both of these null hypotheses can be rejected, one can assume that the effect lies within the range that can be considered negligible.

To determine the upper and the lower boundary of our smallest effect sizes of interest, we constructed two scenarios, in which a significant influence of prior offending behavior on the association of clinically diagnosed pedohebephilia with the VRT index could not be ruled out. A significant influence of prior offending behavior on the association of clinically diagnosed pedohebephilia with the VRT index could not be ruled out if (a) the estimator of the interaction term was small enough to render the association zero or (b) the estimator of the interaction term was large enough to significantly increase the association. For scenario (a), we thus set the lower boundary of our range of possible negligible effects to the lower limit (LL) of the 95%-CI of the simple effect of clinically diagnosed pedohebephilia on the VRT index. For scenario (b), we chose a conservative upper limit of said range of zero. Put otherwise, we deemed any estimator of an interaction effect to be negligible if it were to fall within the boundaries of -LL_95%-CI Pedohebephilia_ and zero, or -LL_95%-CI Pedohebephlia_ < *ß*_*interaction with prior sexual behavior*_ ≤ 0.

Summarizing the above, four null hypotheses had to be examined in order to test Hypothesis 1:

- H0.1: Clinically diagnosed pedohebephilia has a null effect on the VRT index;
- H0.2: The interaction effect of clinically diagnosed pedohebephilia with prior CSO is either greater than zero or smaller than or equal the negative of the lower limit of the 95%-CI of the effect of clinically diagnosed pedohebephilia;
- H0.3: The interaction effect of clinically diagnosed pedohebephilia with prior CSAM offenses is either greater than zero or smaller than or equal the lower limit of the 95%-CI of the effect of clinically diagnosed pedohebephilia;
- H0.4: The interaction effect of clinically diagnosed pedohebephilia with prior CSO and prior CSAM offenses is either greater than zero or smaller than or equal the lower limit of the 95%-CI of the effect of clinically diagnosed pedohebephilia;

We used the 90%-CI of the regression estimates to assess equivalence at alpha = 0.05 (Lakens, 2017).

Hypothesis two was tested using two ROC analyses differentiating (a) individuals with a prior history of CSO with and without self-reported sexual fantasies involving children and (b) individuals without a prior history of CSO or CSAM offenses with and without self-reported sexual fantasies involving children. The resulting areas under the curve (AUC) were tested against the equivalent of random categorization of *AUC* = 0.5. To compare the classification accuracy, we compared the confidence intervals of the AUC. Equivalence on an alpha level of 0.05 was assumed if the 95%-CI of the AUC of individuals with a history of prior CSO encompassed the 90%-CI of the AUC of individuals without a history of prior CSO (Campbell, 2022). Hypotheses 3 to 4a were examined using Spearmann’s rank correlation coefficient rho given the ordinal scales of the behavioral rating tools. Sensitivity analyses were performed on all imputations as well as on data using raw instead of ipsatized viewing reaction time values and including all possible individuals (see Supplement 1 and 2). All analyses were conducted using R-Studio version 1.1.383 (https://rstudio.com) running R statistical software version 3.6.2 (https://www.R-project.org/). ROC analyses were conducted using the package pROC (Robin et al., 2011).

## Results

### Hypothesis 1

: Clinically diagnosed pedohebephilia shows a significant, positive effect on a VRT index regardless of self-reported prior CSO or CSAM.

Using imputation of missing values by individual means per age and sex category, the regression of the VRT index on the clinical diagnosis of pedohebephilia, prior CSO, prior CSAM, and their two- and three-way interaction yielded an adjusted *R*^*2*^ = 34.3%, *F*(7, 274) = 21.95, *p* < 0.0001. Non-pedohebephilic non-offending individuals showed a mean VRT index of *ß* = -1.0945, *SE* = 0.1135, *p* < 0.001. The presence of a clinical diagnosis of pedohebephilia increased the mean VRT index by *ß* = 1.0875, *SE* = 0.2513, *p* < 0.001. Neither a history of CSO, nor of CSAM nor their respective interactions or their interaction with pedohebephilia had an influence significantly different from zero on the VRT index. None of the estimates for the interactions lied within the equivalence boundaries of -LL_95%-CI of ß for_ _pedohebephilia_ ≤ *ß* ≤ 0 with all estimates exceeding the upper threshold of zero and only the estimator of the three-way interaction exceeding the lower threshold of -LL_95%-CI of ß for_ _pedohebephilia_ (see Table 4).

**Table 4:** Regression results using the Viewing Reaction Time Index as the criterion

Predictor	ß	ß—90% CI [LL, UL]
(Intercept)	− 1.0945***	[− 1.2818, − 0.9071]^†^
Pedohebephilia	1.0875***	[0.6727, 1.5023]^‡^
prior CSO	− 0.1192	[− 0.4748, 0.2363]^‡^
prior CSAM offense	0.0087	[− 0.4061, 0.4235]^‡^
Pedohebephilia: Prior CSO	0.2796	[− 0.3111, 0.8703]^‡^
Pedohebephilia: prior CSAM offense	0.0972	[− 0.4675, 0.6618]^‡^
prior CSO: prior CSAM offense	0.4955	[− 0.2828, 1.2737]^‡^
Pedohebephilia: prior CSO: prior CSAM offense	− 0.5741	[− 1.5046, 0.3565]^†‡^

ß represents the regression weight. LL and UL indicate the lower and upper limits of a confidence interval, respectively

^*^p < .05. **p < .01. ***p < .001

^†^marks (LL) + (LL_95%-CI Pedohebephilia_) ≤ 0, ‡ marks UL > 0

Residual standard error: 0.709 on 274 df, Multiple R-squared: 0.3593, Adjusted R-squared: 0.3429, *F*-statistic: 21.95 on 7 df, *p* < 0.0001

Sensitivity analysis showed similar results for all other applied imputation methods with adjusted R^2^ values ranging from 32.8–35.7% and values and direction of the estimators, standard errors, and significance remaining unchanged (see Supplement 1).

Additional analyses using raw instead of ipsatized data and using the maximum available sample found comparable results for the regression analysis, though using raw data, also the estimator of the interaction of clinically diagnosed pedohebephilia with prior CSAM offenses exceeded the lower threshold of our equivalence interval (see Supplement 2).

Testing hypothesis 1 thus yielded inconclusive results.

### Hypothesis 2:

The VRT index separates pedohebephilic and non-pedohebephilic individuals with similar accuracy in those with or without a prior history of sex offenses.

Using imputation of missing values by individual means per age and sex category, the areas under the curve in the two ROC analysis of the VRT index on the clinical diagnosis of pedohebephilia were not significantly different in individuals with (*AUC*_CSO±CSAM_ = 0.8996, bootstrapped 95%-CI = 0.8008–0.9725) and without a history of CSO or CSAM (*AUC*_-CSO-CSAM_ = 0.8231, bootstrapped 95%-CI = 0.6435–0.9538, DeLong’s *D* = 0.91, *df* = 74.01, *p* = 0.4). The 90%-CI of the *AUC*_-CSO-CSAM_ of 0.6667–0.9333 was not encompassed in the 95%-CI of the *AUC*_CSO±CSAM_. An optimal cutoff using the Youden method was determined for the overall sample at a VRT index of -0.5 (Specificity = 75.0%, Sensitivity = 86.4%).

Sensitivity analysis showed similar results for all other applied imputations with *AUC*_CSO±CSAM_ ranging from 0.8947 to 0.9287 and *AUC*_-CSO-CSAM_ from 0.8154 to 0.8423, all DeLong’s tests’ *p*s > 0.5 and none of the 95%-CIs of the *AUC*_CSO±CSAM_ fully encompassing the 90%-CIs of the *AUC*_-CSO-CSAM_ (see Supplement 1).

Additional analyses using raw instead of ipsatized data and using the maximum available sample found comparable results for the ROC analysis (see Supplement 2).

Testing hypothesis 2 thus yielded inconclusive results.

### Hypothesis 3:

Through 4a: Correlations of the VRT index with behavioral markers of pedophilia and re-offense risk

Due to inconsistently missing data in the external criteria, the subsamples available differed for each correlation analysis. Using imputation of missing values by individual means per age and sex category, we found significant positive correlations of the VRT index with the sum scores of the SSPI-2 (*rho* = 0.27, *p* = 0.029), the STABLE-2007 (*rho* = 0.31, *p* = 0.015), ACUTE 2007 (*rho* = 0.34, *p* = 0.007), and the adapted STATIC-99 (*rho* = 0.25, *p* = 0.019). Apart from the factor “General Criminality,” all correlations of the empirical factors of the STATIC-99 and STABLE-2007 were positive, but only the correlation with the STATIC-99 factor “Paraphilia” (*rho* = 0.24, *p* = 0.031) and the STABLE-2007 factor “Sexual Deviance” (*rho* = 0.43, *p* < 0.001) reached significance (Table 5).

**Table 5:** Correlations of Viewing Reaction Time Index with clinical markers of recidivism risk and pedophilia

			VRT index	1	2	3	4	5	6	7	8	9
1	STATIC-PPD	rho	0.1420									
		n	60									
2	STABLE-2007	rho	0.3081^a^	0.4960^d^								
		n	62	52								
3	ACUTE-2007	rho	0.3374^b^	0.3560^b^	0.6104^c^							
		n	62	52	62							
4	SSPI-2	rho	0.2704^a^	0.7206^c^	0.3731^b^	0.2482						
		n	65	58	56	56						
5	STATIC Paraphilia	rho	0.1419	0.8969^c^	0.3214^a^	0.2415	0.8464^c^					
		n	77	60	62	62	65					
6	STATIC Youthful Stranger Aggression	rho	0.1534	0.7730^c^	0.4054^b^	0.1768	0.3412^b^	0.4416^c^				
		n	77	60	62	62	65	77				
7	STATIC General Criminality	rho	− 0.0210	0.5490^c^	0.2142	0.1799	0.1613	0.5512^c^	0.2697			
		n	84	84	62	62	65	84	84			
8	STABLE Antisociality	rho	0.1917	0.4247^b^	0.8572^c^	0.4087^b^	0.2804^a^	0.2560^a^	0.3059^a^	0.2346		
		n	60	49	59	59	53	60	60	60		
9	STABLE Sexual Deviance	rho	0.4704^c^	0.4076^b^	0.5216^c^	0.5345^c^	0.3505^a^	0.1984	0.3010^a^	0.0347	0.2697^a^	
		n	59	48	58	58	52	59	59	59	59	
10	STABLE Hypersexuality	rho	0.2364	0.2996^a^	0.6161^c^	0.4735^c^	0.3258^a^	0.1967	0.3191^a^	0.0185	0.4146^c^	0.4420^c^
		n	60	49	59	59	53	60	60	60	60	59

Coefficients are reported as Spearman’s rho

^a^*p* < 0.05; ^b^*p* < 0.01; ^c^*p* < 0.001

The sensitivity analysis revealed an influence of the imputation method on these correlations. A significant correlation with the STATIC-99 factor “Youthful stranger aggression” was found using predictive mean matching per age and sex category with individuals as separate classes (*rho* = 0.25, *p* < 0.05) and whole sample linear regression per age and sex category (*rho* = 0.22, *p* < 0.05. Using whole sample linear regression per age and sex category also yielded a significant correlation with the STABLE-2007 factor “Hypersexuality” (*rho* = 0.26, *p* < 0.05). Otherwise, the direction and magnitude of the correlation coefficients remained similar across all imputation methods (see supplement 1).

Additional analyses using raw instead of ipsatized data and using the maximum available sample found comparable results for the correlations (see supplement 2).

Hypotheses 3 through 4a were thus supported.

## Discussion

The present study sought to examine the applicability of the VRT paradigm as a diagnostic tool for pedohebephilia in self-referring men from the community, seeking help in a program aimed at the prevention of child sexual offenses. We specifically analyzed the dependence or independence of prior sexual offense behavior and the convergent and divergent validity with behavioral markers of pedohebephilia and sexual re-offense risk.

**Fig. 2:**
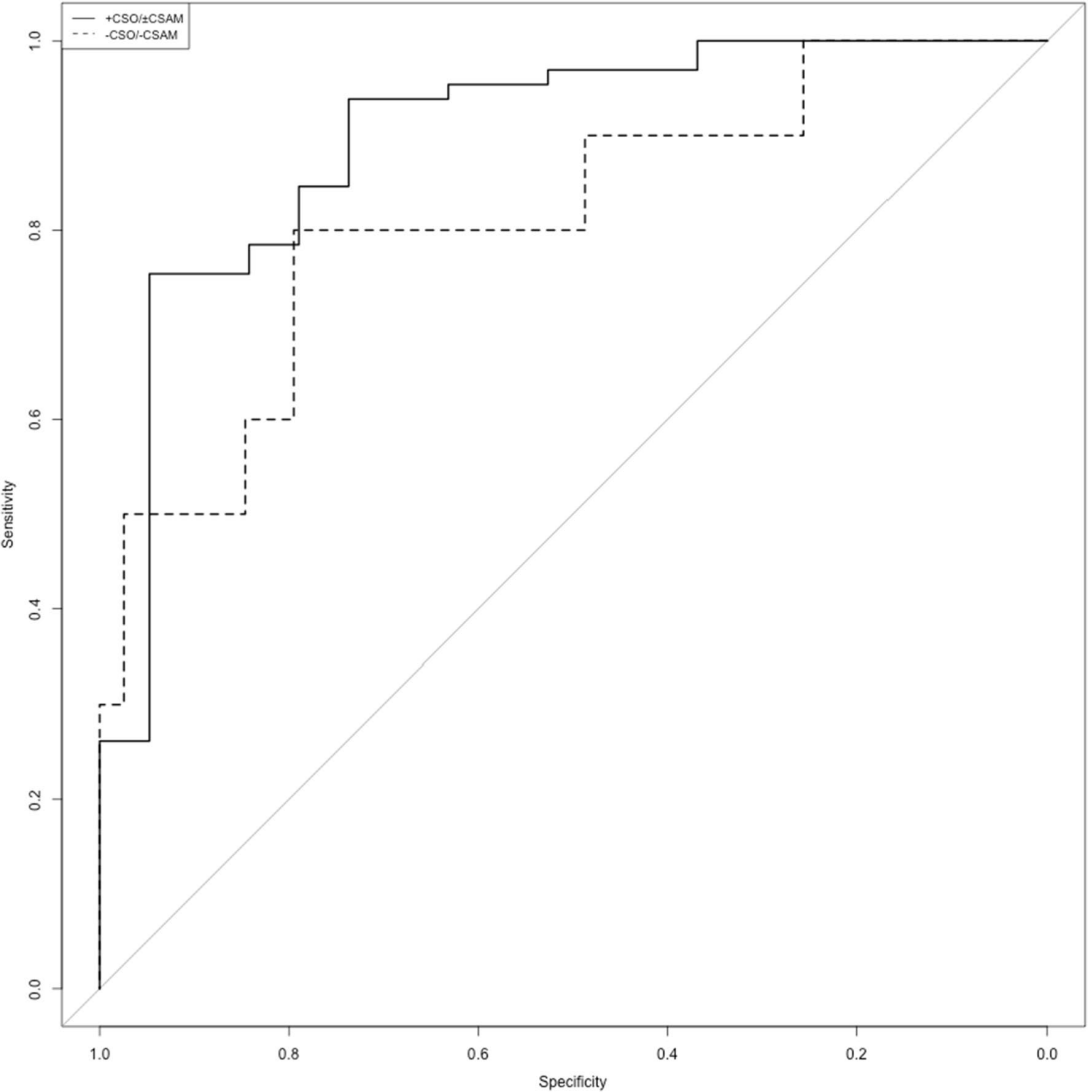
ROC curve for individuals with and without a history of sexual offenses against children. + CSO/ ± CSAM individuals with a history of child sexual offenses, with or without prior use of child sexual abuse materials; -CSO/-CSAM individuals without a history of child sexual offenses or use of child sexual abuse materials

We found the VRT index to be dependent on the clinical diagnosis of pedohebephilia with non-pedohebephilic individuals’ mean VRT index lying around -1 and that of pedohebephilic individuals around zero. Prior CSO and CSAM offenses showed no associations significantly different from zero. Equivalence testing, however, failed to refute a nonzero positive influence on the association of the clinical diagnosis with the VRT index while a non-negligible negative influence on the association was found for the interaction of prior CSO and CSAM offenses with the clinical diagnosis and to some degree the interaction of CSAM offenses with the clinical diagnosis. ROC analyses found no significant difference in the discriminative ability of the VRT index in individuals with and without a history of sexual offenses against children. Equivalence testing, however, failed to refute negligible differences. An optimal cutoff using the Youden method was determined for the overall sample at a VRT index of -0.3 (Specificity = 82.7%, Sensitivity = 79.3%) (Fig. 2).

The VRT index showed no correlation with an adapted STATIC-99 score while the correlations with the SSPI-2, the STABLE-2007, and the ACUTE-2007 scores were small and significant. Analyses of the empirical factors of the STATIC-99 and STABLE-2007 found significant positive correlations only with factors indicating a potentially problematic sexual motivation such as “Deviant Sexuality” and “Hypersexuality” of the STABLE-2007 but not with factors associated with other criminogenic needs such as “Antisociality.”

Our data constitute the second time the association of the viewing reaction time to visual stimuli of children and prior overt sexual behavior with children has been examined in individuals with pedohebephilia. Jahnke et al. (2022) found no significant effect in non-offending individuals with sexual interest in minors before but provided no tests of equivalence. Also in their population, the independent variable was self-identification rather than clinically assessed sexual preference leaving more room for self-deception and social desirability, and their study only considered past convictions for sexual offenses, whereas our data included any prior sexual offending behavior against children, both detected and undetected. While our study thus corroborates the finding of Jahnke et al. (2022), it provides evidence over and above their data: Similar to their findings, our study did not show a significant association of either convicted or undetected prior sexual behavior with children and the VRT effect. Our equivalence tests, however, were unable to rule out a small positive association in all prior behaviors and a non-negligible negative association of prior CSAM offenses or CSAM offenses and CSO combined. The relevance of the potential positive association is difficult to gauge. With the mean difference between non-pedohebephilic and pedohebephilic individuals of roughly one standard deviation, our chosen upper threshold for the equivalence interval of zero can be considered conservative and the violations of that upper threshold might prove irrelevant after all. On the other hand, an increased difference of the VRT index between individuals with and without a clinical diagnosis of pedohebephilia in individuals with prior CSO offenses might speak to a greater reliability of the instrument in this population. This interpretation finds further support with the equivalence tests in the ROC analysis failing to refute similarity of the AUC of individuals with and without prior CSO. The failure to establish a statistically significant difference between the two AUCs might thus rather be a problem of a lack of statistical power than indicating actual similarity.

Our equivalence tests showed a non-negligible negative effect of the interaction of prior CSO and CSAM offenses with the clinical diagnosis of pedohebephilia on the VRT index. Moreover, our sensitivity analyses found a potentially mitigating effect of the interaction of prior CSAM offenses alone with the clinical diagnosis in the model using raw VRT data. This finding needs to be interpreted with caution, as the estimator of the three-way interaction of CSO: CSAM: Pedohebephilia producing the more stable violation of the lower boundary was also the one with the largest error margin, whereas for the two-way interaction of CSAM: Pedohebephilia, only the estimator for the raw viewing reaction time latencies exceeded the lower boundary of -LL_95%-CI ß Pedohebephilia_. With that caveat in mind, this potential negative effect is easier to interpret. Our lower bound of the equivalence interval was chosen so that any interaction effect falling below this threshold could potentially nullify the association of the clinical diagnosis and the VRT index. In other words, the results of our equivalence tests indicate that in individuals with prior CSAM offenses alone or in conjunction with prior CSO offenses, the association of the clinical diagnosis of pedohebephilia and the VRT index might be virtually zero. The absence of such an association in a prior study applying a classical null-hypothesis testing logic (Rosburg et al., 2021; Schmidt et al., 2014) does not preclude its existence as rejecting the alternative hypothesis must not be taken as proof for the null hypothesis (Lakens, 2017). Also, research in teleiophiles indicating the independence of the VRT effect from prior behavior (Imhoff et al., 2012) may not translate readily to pedohebephilic populations, given that teleiophilic sexual interests are far more likely and far less consequential to be lived out. In teleiophilic populations, a comparable design to the one at hand would consist in comparing completely celibate individuals with exclusive porn users and individuals with experiences of either or both pornography use and actual sexual contact. Such examinations appear to be missing.

A potential null association between the clinical diagnosis and the VRT index in these populations, i.e., CSAM offenders and offenders committing both, CSAM offenses and CSO, merits further interpretation. Following the interpretation of Schmidt et al. (2017), the VRT effect stems from the difference between rejecting clearly irrelevant stimuli and scrutinizing potentially relevant sexual stimuli. Offenders committing CSAM offenses typically show high frequent use of the images and should thus show enhanced recognition of sexually relevant features of the presented images. Such enhanced recognition might lead to the non-negligible mitigation of the VRT effect. Other explanations seem also possible. Clinicians might bear a specific bias in these populations and wrongly diagnose pedohebephilia, given that both behavioral constellations, i.e., CSAM offending and the so-called “mixed” offending, have been associated with a greater probability of a genuine sexual interest in children (Babchishin et al., 2015). Also, non-exclusive pedohebephilic interests, i.e., in addition to sexual interest in adults, might be more prevalent in these two populations as indicated by greater frequencies of marriage compared to populations who exclusively committed CSO (Babchishin et al., 2015).

The inspection of the descriptive VRT data and the *ß*-estimates supports this interpretation. The intercept of roughly -1 indicated that the mean expected response latency of individuals without pedohebephilia or a history of child sexual offenses toward adult stimulus categories was one standard deviation larger than that toward children. With the *ß*-estimate for pedohebephilia being approximately + 1, on the other hand, our data indicated that in individuals with pedohebephilia, the expected mean difference between response latencies was approximately zero. In other words, the VRT effect in our study was driven by individuals with pedohebephilia showing no difference in response latencies toward child and adult stimuli, whereas individuals with teleiophilic interests showed greater response latencies toward adults than toward child stimuli. A decreased differentiation in responding to adult and child sexual stimuli in individuals with pedohebephilia somewhat contrasts work in phallometry, arguing that the relatively stronger sexual response to child stimuli in comparison with the response to adult stimuli should be seen as indicative of pedohebephilia (Blanchard et al., 2009). However, a recent meta-analysis found that presumably pedophilic sex offenders against children did not show statistically significant differences between their sexual interest in children and in adults across a number of psychophysiological markers (Schippers et al., 2023). The reasons for this remain unclear. With the VRT effect depending on cognitive, self-evaluative rather than “hot” affective processes, a possible interpretation of this finding lies in the clinical, help-seeking population, where a common concern voiced reads “Am I pedophilic?” Such concerned individuals might be prone to increased scrutiny while evaluating the arousing potential for themselves of stimuli of both adults and children. Such interpretation would also fit with observations in a community sample showing sex guilt to be correlated negatively with the viewing reaction time effect (Love et al., 1976). Another possible explanation lies in the common coexistence of multiple sexual age preferences including coexisting preferences for mature and immature developmental stages (Beier et al., 2013). With only 52 of the pedohebephilic sample reporting an exclusive attraction toward minors, the coexisting attraction toward adults in the other 169 individuals with pedohebephilia may have had an attenuating effect on the VRT index. Additionally, on a psychophysiological level, penile responding to stimuli of adjacent age and sex categories (e.g., females or males in Tanner stages 2, 3, and 4) have been found to follow a pattern similar to that of a stimulus generalization gradient (Blanchard et al., 2010; Frenzel, 1990). A sizable proportion of individuals reported hebephilic sexual interests in our sample, i.e., interest in children in developmental stages 2 and 3 according to Tanner (1973). Following the stimulus generalization gradient described above, the next greatest psychophysiological responses in these individuals are to be expected toward stimuli in Tanner stage 4. The finding of equal viewing reaction times to stimuli of mature and immature individuals might represent this stimulus generalization gradient. Analyzing these potential effects, however, was beyond the scope of the present study.

The analysis of the convergent and divergent validity of the VRT effect in our sample of self-referring individuals with prior sex offenses against children yielded negligible to small positive correlations with behavioral markers of pedohebephilia and re-offense risk. The range of these correlations lied well within that of a prior meta-analysis (Pedneault et al., 2021). There, the correlation of the VRT with the SSPI was reported with *r* = 0.15 across seven studies where the 95%-CI included the zero and of 0.20 across five studies with markers of the offense history where the 95%-CI did not include zero. Furthermore, our correlation with the SSPI-2 was similar to the *r* = 0.27 reported in its original developing study with the psychophysiological gold standard of phallometry. These low correlations reflect the significant but only loose connection of the sexual preference for minors and child sexual offending. The SSPI-2 counts the number of certain overt sexual behaviors involving children to gauge the likelihood of an underlying pedohebephilic sexual preference. However, neither do individuals with pedohebephilia necessarily commit multiple sexual offenses against children nor are serial offenses restricted to this population. The adapted STATIC-99, whose “Paraphilia” factor comprises some items reflected in the SSPI-2, showed the smallest correlations with the VRT effect. Major caution needs to be taken when interpreting this finding, as our measure was devised as a provisional adaptation to the Dunkelfeld. However, given the similarities with the SSPI-2 items, the small correlation is unsurprising.

We found the significant correlation of the VRT effect with the STABLE-2007 to be driven by the factors “Sexual Deviance” and “Hypersexuality.” “Hypersexuality” or an increase of sexual behavioral outlets thereby has been found to be a frequent feature of the paraphilias in general and in individuals with pedohebephilia specifically (Gerwinn et al., 2018; Kafka & Hennen, 2002). The correlations with factors of the STABLE-2007 associated with deviant sexuality (convergent validity) but not with antisociality (divergent validity) thus corroborate the relevance of the VRT as a diagnostic tool for the motivating sexual preference rather than facilitating antisociality (Seto, 2017).

The significant correlation with the ACUTE-2007, however, is not readily interpretable. Such association also stands in contrast with prior findings (Schmidt et al., 2017). A possible explanation lies in the specifics of the program from which the sample was recruited. This program’s public relation work has an emphasis on the risk for child sexual offenses as represented in the English website’s name “dont-offend.org.” This emphasis may lead to an over representation of individuals in precarious circumstances, perceiving themselves at a higher risk to act out on their sexual preference. The aspects of psychosocial destabilization as measured by the ACUTE-2007 might thus be associated with a motivation to seek therapeutic help in individuals with pedohebephilia outside the forensic context.

### Limitations

As one potential application of the VRT lies in its use to support a clinical diagnosis of pedohebephilia, the clinical setting in which our data were gathered provides good ecological validity of our analysis. At the same time, one important limitation for the generalization might be seen in the procedure of taking the VRT after an extensive focused sexual diagnostic interview. With the VRT effect evidently depending on cognitive processes, an effect of this interview on the participants’ behavior during the VRT is plausible but could not be quantified in our study. Also, by excluding all diagnostically unclear cases from the sample we used a rather progressive test of the validity of the VT measure which may preclude generalization to a clinical setting.

The lack of objective verification of the self-reported sexual offenses may have introduced a separate bias. Within our sample of individuals without diagnosable prior offenses, some may have been able to hide their prior offending from the examiners and thus been falsely assigned to the non-offending group. Furthermore, the number of non-offending individuals with pedohebephilia was quite low. Both the findings concerning the association of the VRT effect with prior overt sexual behavior with children and its discriminatory power may have been affected by these limitations. Our results should thus be viewed as preliminary until further corroboration.

As mentioned above, none of the instruments for re-offense risk assessment have been validated in the Dunkelfeld. The STATIC-99 is so very dependent on forensic markers that the instrument as a whole needed to be adapted to be used in our population. Also, their development for convicted sexual offenders rendered them dependent on prior CSO impeding their applicability for non-offending individuals. Our findings concerning these instruments therefore need to be interpreted with caution in that a generalization to offending individuals in forensic contexts cannot be assumed with certainty and a validation with external criteria in individuals without prior CSO remains pending.

### Conclusion

Our study provides first evidence for the validity of the VRT effect to detect pedohebephilia in a clinical sample outside the forensic context. Classical null-hypothesis testing found no significant influence of prior behaviors on the VRT effect. With an equivalence testing approach failing to refute non-negligible positive influences of prior CSO and both non-negligible positive and negative influences of prior CSAM offenses, further investigations into possible mediating effects of overt sexual behavior on the VRT effect are warranted. In a subsample of individuals who reported prior CSO, we found evidence for convergence with behavioral markers of pedohebephilia while markers of antisociality were uncorrelated. An additional correlation with markers of imminent risk, namely hypersexuality and psychosocial destabilization, may speak to the specific needs of a clinical population. With non-offending individuals with pedohebephilia remaining a vastly understudied population though potentially crucial in order to understand roots and consequences of this special sexual interest, our study suggests that the VRT might provide an objective method to discern individuals with and without pedohebephilia outside a forensic context irrespective of their offense history.
